# Acute renal failure following consumption of fish gall bladder

**DOI:** 10.4103/0971-4065.59339

**Published:** 2009-10

**Authors:** P. C. Bhattacharyya, M. Nayak, A. Barkataky

**Affiliations:** Down Town Hospitals, Guwahati, Assam

**Keywords:** Acute kidney injury, acute tubular necrosis, fish gall bladder

## Abstract

A case of acute renal failure developing after consumption of fish gall bladder as a food item is reported. The patient recovered fully with conservative treatment and dialysis. The risk of acute kidney injury following ingestion of fish gall bladder, apparently for medical reasons is highlighted.

## Introduction

Consumption of fish's gall bladder as a food item is common amongst the non-vegetarian people of Assam, a North Eastern State of India. Many people of this region believe that fish's gall bladder of grass carp variety (*Ctenopharyngodon idellus)* as food is good for health, improves vision and cures rheumatism. Chinese people believe that fish's gall bladder cures fever and asthma.[1] However, at times this may cause toxic hepatitis and acute renal failure.[1,2] Poisoning due to ingestion of fish gall bladder was first reported from China. A syndrome of ARF and acute hepatitis was also reported from Hongkong,[3] Japan,[4] India[5] and United States.[6] Recently, three more cases were reported from Manipur, India.[7] The mortality and morbidity rate following ingestion of gall bladder of fish is significant. Acute renal failure occurs in 50-100% of all fish gall bladder poisoning and fish gall bladder poisoning accounts for 91.7% of the mortalities due to ARF.[8] Because of its clinical significance, we report a case of ARF following consumption of grass carp gall bladder in an young female nurse of our hospital.

## Case Report

A 23-year old staff nurse was admitted in down town hospital, Guwahati, Assam on 21^st^ March 2004 with a history of loose motion, nausea and vomiting since 2 days. Reportedly, she had consumed gall bladder of grass crap fish with her regular food 48 h before the development of symptoms. There was no history of consumption of any other food item or drug. Physical examination on admission revealed moderate dehydration, mild pallor, but no pedal oedema. The pulse rate was 94 bpm, blood pressure 140/80 mm of Hg, temperature 97.8°F, respiratory rate was 20 per min, systemic examination was normal. Laboratory examination revealed T C-7400/cu.mmm; P_68_, L_27_, M_2_, E_3_, ESR- 10; Hb-11.0 gm, S. creatinine 3.4 mg/dl; urea- 110 mg/dl; S. protein level- 7.5 mg/dl; Serum Na^+-^ 137 meq/l; Serum K^+^ 4.1 meq/l; S. calcium 8.1 mg/ dl; S. amylase 38 mg/dl; S. lipase 46 mg/dl; urine routine examination revealed presence of albumin. Blood sugar level, LFT, serum lipid profile, ECG, plain radiograph of KUB, chest X-ray, renal ultrasound all were normal. Kidney biopsy was not done. She was managed conservatively for ARF and other clinical problems. On the second day, diarrhea was controlled but urine output came down, nausea and vomiting persisted. Repeat renal function tests revealed further elevation of serum creatinine (6.5 mg/ dl), Urea (108 mg/dl), S k^+^ 5.5 meq/l and S Na^+^ 145 meq/l. Patient was put on hemodialysis on the third day of hospitalization. She required 3 sittings of hemodialysis and on the eighth day after third haemodialysis her blood urea came down to 60 mg/dl, S. creatinine 3.0 mg/dl, S Na^+^ 137 meq/l, S k^+^ 4.0 meq/l. She showed signs of improvement, diuretics were started, and was discharged on the twelfth day with the normal urine out put with normal electrolyte levels.

## Discussion

Poisoning leading to acute kidney injury following injection of gall bladder of grass carp has been reported from different parts of the globe.[3–7] Patients usually present with pain abdomen, nausea, vomiting, loose motion and develop ARF and hepatitis. Renal failure may be oliguric or non-oliguric and usually develop on second or third day of consumption of gall bladder of fish. The present case also developed all symptoms including renal failure after 48 h of intake of the food, showed gradually deteriorating renal failure. Among fish gall bladder poisoning, ciguatera and scombroid poisoning are the most commonly recognized. However the raw gall bladder of the grass carp (*Ctenopharyngodon idellus*) with both nephrotoxic and hepatotoxic properties is less known.[9] Recently, studies have shown that fish gall bladder can also damage the heart, liver, gastro-intestinal tract and lead to multiple organ dysfunction syndrome (MODS) in addition to ARF.[9] Death due to poisoning following ingestion of fish gall bladder was reported from Vietnam[10] also. ARF may be caused by nephrotoxicity of bile, or pre-renal factors like hypotension, volume depletion leading to ATN.[10,11] In our patient the most likely cause of ARF was nephrotoxicity of bile. She did not have any evidence of other factors. Cyprinol, a bile alcohol found in bile of common carps, may be the cause of hepatic and renal toxicity.
